# Immune response from a resource allocation perspective

**DOI:** 10.3389/fgene.2012.00267

**Published:** 2012-12-14

**Authors:** Wendy M. Rauw

**Affiliations:** Departamento de Mejora Genética Animal, Instituto Nacional de Investigación y Tecnología Agraria y AlimentariaMadrid, Spain

**Keywords:** life history theory, resource allocation, selection, immune function, tolerance, resistance, robustness

## Abstract

The immune system is a life history trait that can be expected to trade off against other life history traits. Whether or not a trait is considered to be a life history trait has consequences for the expectation on how it responds to natural selection and evolution; in addition, it may have consequences for the outcome of artificial selection when it is included in the breeding objective. The immune system involved in pathogen resistance comprises multiple mechanisms that define a host's defensive capacity. Immune resistance involves employing mechanisms that either prevent pathogens from invading or eliminate the pathogens when they do invade. On the other hand, tolerance involves limiting the damage that is caused by the infection. Both tolerance and resistance traits require (re)allocation of resources and carry physiological costs. Examples of trade-offs between immune function and growth, reproduction and stress response are provided in this review, in addition to consequences of selection for increased production on immune function and *vice versa*. Reaction norms are used to deal with questions of immune resistance vs. tolerance to pathogens that relate host health to infection intensity. In essence, selection for immune tolerance in livestock is a particular case of selection for animal robustness. Since breeding goals that include robustness traits are required in the implementation of more sustainable agricultural production systems, it is of interest to investigate whether immune tolerance is a robustness trait that is positively correlated with overall animal robustness. Considerably more research is needed to estimate the shapes of the cost functions of different immune strategies, and investigate trade-offs and cross-over benefits of selection for disease resistance and/or disease tolerance in livestock production.

## Introduction: immune function is a life history trait

Life history theory deals with the way an organism spreads its reproduction over its lifetime and forms an adaptation to the environment it lives in (Brommer, 2000; Van Straalen and Roelofs, 2006). It is commonly defined as a set of evolved behavioral and physiological strategies that more or less influence longevity and reproduction and may include fitness traits such as reproductive success, survival, viability, fecundity, mating success, and age at maturity (Schluter et al., 1991; De Jong, 1994; Ricklefs and Wikelski, 2002). In the absence of trade-offs, natural selection would drive all life-history traits to limits imposed by animal design, where the evolutionary ideal would be an organism that matures upon birth and reproduces non-stop, producing clones of itself and never dying. However, a fundamental assumption of life history theory is that resources are limited and need to be invested amongst growth, reproduction, and maintenance, or stored for future use, and since resources used for one purpose are no longer available for other purposes, trade-offs are inevitable (Leroi, 2001; McDade, 2005; Van Straalen and Roelofs, 2006; Roff, 2007). Natural selection results in the optimal allocation of resources across important life history functions and prunes away less-optimal strategies (Brommer, 2000): “The vigorous, the healthy, and the happy survive and multiply” (Darwin, 1872).

Although the majority of life history studies focus on factors related to reproduction and growth, fitness does not only depend on reproductive success, but also on maintenance of existing structures and longevity (Lochmiller and Deerenberg, 2000). The immune system is a major physiological system centrally involved in cellular renewal and repair, and as such, it is an essential component of body maintenance (McDade, 2005). Parasites and pathogens are the greatest threat to survival by most animals, where the immune system is the major physiological mechanism regulating host survival (Lochmiller and Deerenberg, 2000). Therefore, the immune system is a life history trait which can be expected to trade off against other life history traits according to theory. These trade-offs are likely to influence not only how vigorously an organisms defends itself, but also which of the parts of the immune systems are emphasized (Lee et al., 2008).

Whether or not a trait is considered to be a life history trait has consequences for the theory on how it responds to natural selection and evolution. In addition, it may have consequences for the outcome of artificial selection when it is included in the breeding objective. According to the Resource Allocation Theory developed by Beilharz et al. (1993), when the amount of resources increases (because of a favorable environment) these resources will be used by the organism to raise fitness: “organisms respond to natural selection until fitness can improve no more. That is the point at which organisms utilize all available resources of the environment most efficiently.” Selection for high fitness by natural selection will lead to intermediate optimal values for the fitness components and heterozygosity which imparts a buffering capacity to a wide range of environments (Dunnington, 1990; Beilharz et al., 1993). However, animals that originate from a population selected for a trait requiring resources may preferentially allocate resources to this trait, reducing the availability of resource to respond to other demands. Rauw et al. (1998) and Rauw (2007) showed that the highly favorable increase in production levels in broilers, pigs and dairy cattle is often compromised by behavioral, physiological, and immunological problems. Likewise, increasing energy expenditure on maintenance related traits may reduce the availability of resources for production. When artificial selection for immune function is considered, it is of interest to evaluate the costs and possible trade-offs of immune mechanisms. This is the aim of this review.

## Immune function: resistance vs. tolerance

### Resistance

The immune system involved in pathogen resistance comprises multiple complementary, interdependent subsystems that either prevent pathogens from invading, or eliminate the pathogens when they do invade, i.e., they directly reduce the reproductive potential of the pathogen and limit the pathogen burden (Roy and Kirchner, 2000). The innate, non-specific defenses recognize antigens that are general to a wide range of pathogens and entail a series of actions that transpire almost immediately after recognition of an invading pathogen (Janeway and Medzhitov, 2002; Kogut, 2009). All multicellular organisms have some kind of innate defense; roughly 98% of all multicellular organisms possess only an innate immune system for protection against infections (Kogut, 2009). The costs of constitutive innate immunity have not been definitively measured, but the developmental costs are thought to be comparatively low because of the lack of a diversification process, low rates of cell turnover when an immune response is not being mounted, and the small tissue mass accounted for by the cells and proteins involved in the innate response (Lee, 2006). However, the constitutive components of the innate immune system can induce local inflammation via the production of inflammatory cytokines, and if highly stimulated induce the highly costly systemic inflammatory response, which is characterized by increased production of acute phase proteins by the liver, changes in energy and nutrient metabolism, anorexia and fever, leading to localized tissue damage and potentially sepsis (Cohen, 2002; Lee, 2006; Kogut, 2009).

The adaptive, specific immune defenses utilize receptors on T and B lymphocytes that recognize specific antigens on pathogens with great precision. They are characterized by an enormous range of diversity in antigen-binding receptors and have the ability to recognize and respond more quickly to antigens upon second exposure through immunological memory (McDade, 2005; Bowden et al., 2007). They are generally divided into cell-mediated and humoral components. Cell-mediated immunity (type one T-helper cells and cytotoxic T-lymphocytes) primarily defends against intracellular pathogens such as viruses, and similar to induced innate immunity, cell-mediated responses are accompanied by the secretion of proinflammatory cytokines and are sometimes associated with the nutritionally expensive systemic inflammatory response (Lee, 2006). In addition, the rapid expansion of T-cells during development and later diversification require substantial time and nutrients (Lee, 2006). An estimated 95% of maturing T cells is destroyed in the thymus as a result of rigorous selection procedures, making this a very expensive process (McDade, 2005). The costs of using the humoral component (B-cells and type two T-helper cells) are thought to be small compared with those of innate and cell-mediated defenses because the humoral immunity is associated with the production of anti-inflammatory cytokines; however, lymphocyte proliferation and diversification during the developmental period require substantial energy and nutrients (Lee, 2006). The adaptive defense is of more recent evolutionary origin and occurs in jawed vertebrates, although highly discriminatory defense responses have been identified in a number of invertebrate groups, suggesting that pathogen-specific responses might have evolved in numerous occasions and that disease-specific immunity might be commonplace in the animal kingdom (Råberg et al., 2002; Bowden et al., 2007). Immune responses mediated by T and B cells are protective to the host, but may become deleterious when immune reactions are misguided or excessive, resulting in serious damage to the host from autoimmunity or allergy (Sakaguchi et al., 2008).

Activation of the innate response is generally considered to be more costly than activation of the adaptive response (Lee et al., 2008; Colditz, 2009; Sykes, 2010). However, during re-exposure of the host to pathogens there may still be activation of innate immune pathways such that adaptive immunity may not be able to circumvent all the costs of innate immune responses (Colditz, 2009).

### Tolerance

A second type of defense is pathogen tolerance, literally meaning “a change in sensitivity to an immune elicitor” (Ayres and Schneider, 2012). Tolerance involves limiting the damage that is caused by the infection and does not involve inhibiting pathogen growth or reproduction (Roy and Kirchner, 2000). Whereas much is known about the mechanisms involved in pathogen resistance, a systematic understanding of pathogen tolerance is limited, particularly in animals (Råberg et al., 2007; Schneider and Ayres, 2008). Tolerance is a concept that is not tied to one particular physiological mechanism (Ayres and Schneider, 2012). Schneider and Ayres (2008) consider three classes of mechanisms that can affect tolerance:

Effector molecules that induce resistance mechanisms that can cause self-harm and as a result decrease tolerance. Tolerance to the damage caused by pathogens includes all of the mechanisms employed to regulate self-harm caused by aberrant immune responses (i.e., pathogen resistance mechanisms), such as autoimmunity or allergy.
Signaling molecules that activate immune cells that do not cause pathology directly but may decrease tolerance through the damage induced by effectors of the activated immune cells as well as additional pathology caused by other targets of the signaling molecules.
(a) Toxic compounds produced by the host or pathogen resulting in damage to the host; (b) resistance responses that require a high level of energy expenditure leaving fewer resources available for repair of damage to other systems; (c) physiological changes induced by immune responses that are deleterious for other systems; (d) repair of tissue damage; (e) evolution of pathogen-specific solutions to infection.

In addition, interactions with mutualistic and commensal bacteria might reveal more tolerance mechanisms, including those encoded by pathogens themselves (Schneider and Ayres, 2008). Based on these classes of mechanisms, tolerance may be increased in a number of ways through damage prevention and damage repair. Firstly, by actively blocking immune detection, by lacking receptors that recognize a benign/mutualistic microbe, by keeping an immune response switched off until needed, or by (locally) reducing the activation of resistance mechanisms or selectively blocking specific signaling pathways. Secondly, by reducing self-harm resulting from the activation of resistance mechanisms, such as with having a higher affinity for pathogen-associated molecules than for self-molecules, or resulting from the elimination of self-reactive T-cell receptors and antibodies. Thirdly, by maintaining a sufficient resource intake and resource allocation, and fourthly by increasing tissue repair if pathology cannot be entirely prevented (Schneider and Ayres, 2008; Ayres and Schneider, 2012).

It is the sum of resistance and tolerance that defines a host's defensive capacity and both are genetically determined by many genes that affect different components of the immune system (Warner et al., 1987; Schneider and Ayres, 2008). The diverse immune responses are context specific and the costs will vary with the pathogen, the environment, resource availability, the developmental stage of the host, and the genotype of the host (Sandland and Minchella, 2003; Colditz, 2009).

## Metabolic costs of the immune response

### Resistance

Immune defenses are energetically expensive; therefore, the rate at which organisms transform energy and nutrients can be expected to be elevated as a result of immune defense activation. Infection, trauma, and injury may result in a stereotypical response that includes loss of appetite, increased sleepiness, muscle aches, and fever. Fever, characterized by an adaptive increase in the set point for body temperature, is a complex, coordinated autonomic, neuroendocrine, and behavioral adaptive response which is used by nearly all vertebrates as part of the acute-phase reaction to immune challenge (Saper and Breder, 1994; Kluger et al., 1998). It has been associated with improved survival and shortened disease duration in non-life-threatening infections (Hasday et al., 2000). Fever is energy intensive, entailing an increased metabolic cost (Baracos et al., 1987; Nilsson, 2003). Depending on the species, fever requires a 7–15% increase in caloric energy production for each degree Celsius of increase in body temperature (Elia, 1992; Demas et al., 1997; Nilsson, 2003). In order to meet the accelerated rates of caloric expenditures associated with fever, the body must depend primarily on its stores of metabolizable energy (Beisel, 1977).

Metabolic rate in infected animals has been mostly investigated in small mammals and birds. Demas et al. (1997) showed that adult mice immunized with keyhole limpet hemocyanin (a relatively mild antigen that causes limited activation of the immune system) expended significantly more O_2_ than control mice injected with saline and suggested that the energetic costs assessed in their study would be greatly increased with the use of more ecologically relevant antigentic challenges, such as bacteria or parasites. Mounting an immune response in male great tits injected with sheep red blood cells resulted in nearly 9% higher basal metabolic rates in the study of Ots et al. (2001). In addition, the animals also lost nearly 3% of their body mass subsequent to the immune challenge. In the study of Nilsson (2003), mass-specific resting metabolic rate, measured during the night when animals were inactive, was 17% higher for flea-invested marsh tit nestlings compared to control nestlings; nestlings have to depend on their innate immune system to take care of antigens. House sparrows injected with phytohaemagglutinin, a commonly used mitogen that activates the cell-mediated immune response, increased their resting metabolic rate with 29%. It was concluded that immune activity in wild passerines increases energy expenditure, which in turn may influence important life-history characteristics such as clutch size, timing of breeding or the scheduling of moult (Martin et al., 2003). Subsequent to immune challenge with nylon implant, white cabbage butterfly pupae increased their standard metabolic rate by nearly 8% compared to controls; this study was the first direct evidence indicating that activation of the immune system is energetically costly in insects (Freitak et al., 2003). According to Derting and Compton (2003), the cost of maintaining the immune system is minimal in wild white-footed mice (*Peromyscus leucopus*), but in contrast, there is a significant energetic cost of mounting an immune response.

Other immune activities related to pathogen resistance that require energy include the change in size and rate of turnover of cell and protein pools of the immune system; many components of the immune effector responses are highly proteinaceous in nature (Kyriazakis and Houdijk, 2006; Segerstrom, 2007; Colditz, 2009). Barnes et al. (2002) observed an increased fractional rate of protein synthesis of 141% in liver, 161% in plasma, and a 266% hemopexin fractional synthesis rate after injection with *Escherichia. coli* lipopolysaccharide in chickens. Some studies have attempted to quantify these costs experimentally. For example, Yewdell (2001) considered the overall protein economy of cells in relation to protein folding, ubiquitin-targeted proteasome-mediated degradation of proteins and the generation of peptide ligands for major histocompatibility complex (MHC) class I molecules, and Princiotta et al. (2003) quantified the macroeconomics of protein synthesis and degradation and the microeconomics of producing MHC class I associated peptides from viral translation products.

### Tolerance

Protein turnover is also involved in immune tolerance in tissue replacement and repair when damage cannot be prevented during infection. For example, mastitis, an inflammatory reaction of the mammary gland that is usually caused by a microbial infection results in tissue damage induced by either apoptosis or necrosis where both bacterial factors and host immune reactions contribute to epithelial tissue damage (Zhao and Lacasse, 2008). Larvae of several common species of parasitic nematodes migrate through, and often damage, host lungs (Hoeve et al., 2009). The wound is a site of intense metabolic activity characterized by dissolution and removal of necrotic tissue, containment and killing of pathogens, collagen and elastin synthesis and wound repair, cellular proliferation, and restoration of tissue integrity, requiring both energy and substrates (Bessey, 2004). Following injury, there is increased activity of protein, carbohydrate and fat-related metabolic pathways and of many ion pumps, and an increased blood flow to the damaged tissue (Bessey, 2004; Walsh, 2007). Increased protein turnover and accelerated muscle protein breakdown resulting in muscle wastage serves to mobilize amino acids for synthesis of new protein in wounds, for proliferation of phagocytes, macrophages, and other cellular components involved in wound healing, and for synthesis of acute-phase proteins and glucose in the liver (Bessey, 2004).

The deployment cost occurring when the immune system responds can be measured as an increase in metabolic activity because it uses up tangible parts of an organism's energy budget. However, the costs of maintenance functions in response to tissue damage are intrinsically difficult to measure and difficult to separate from other cell maintenance functions that are not part of the immune function (Schmid-Hempel, 2003). Consequently, little is known about the actual resource costs of immune tolerance. Repeated breakdown and resynthesis of proteins in cycles that use energy for no apparent net gain are costly and may appear to be energetically wasteful and futile. For example, if protein accretion would involve digestion, absorption, transport, uptake, and synthesis, net efficiency would fall in the range of 75–85%; turnover can reduce this efficiency by 15–40% (Baldwin et al., 1980). However, protein turnover provides the flux that is necessary for metabolic regulation and adaptation (Hawkins, 1991). The cost of tissue repair depends on the level of damage, as the larger the wound, the more intense the metabolic responses (Bessey, 2004).

## Evolution of immune mechanisms

Evolution has led to a variety of defense mechanisms; however, a universally perfect defense has not evolved. Two lines of theories may explain the existence of variation in the success of defense. Firstly, pathogens or parasites usually evolve faster than their hosts where pathogens and parasites continuously track host defenses and evolve to bypass them (Jokela et al., 2000). Mechanisms employed by the pathogen that determine their virulence and mechanisms employed by the host to protect themselves result in parasite-mediated evolution of host phenotypes, resulting in an extremely complicated protection machinery (Roy and Kirchner, 2000; Freitak et al., 2003; Møller and Saino, 2004; Svensson and Råberg, 2010). As Haldane (1949) stated “the most that the average species can achieve is to dodge its minute enemies by constantly producing new genotypes” (in Duffy and Forde, 2009).

Employing resistance vs. tolerance mechanisms may have different consequences for the coevolutionary interactions between hosts and pathogens because of the differential consequences that these two mechanisms may have on the fitness of each (Møller and Saino, 2004; Svensson and Råberg, 2010). Theoretically, tolerance mechanisms, in compensating for damage, will increase pathogen fitness and therefore disease prevalence, resulting in an evolutionary advantage of carrying tolerance genes, driving them to fixation by selection. In contrast, by inhibiting infection, resistance mechanisms reduce pathogen fitness where the subsequent reduced disease prevalence will reduce the advantage of carrying resistance genes, which therefore cannot become fixed (Roy and Kirchner, 2000; Best et al., 2009). Plant studies suggest that tolerance and resistance might be mutually redundant, such that selection for tolerance in hosts should reduce selection for resistance, and vice versa (Svensson and Råberg, 2010). Indeed, in the study of Råberg et al. (2007), resistance and tolerance were negatively genetically correlated in laboratory mice infected with rodent malaria. However, Mauricio et al. (1997) suggest that both tolerance and resistance may coexist stably in populations of the plant species *Arabidopsis thaliana*, calling into question the likelihood of mutual exclusivity suggested by other authors. The latter was supported by a study of Fornoni et al. (2004), who indicated that variable costs and benefits of tolerance and resistance can result in the maintenance of intermediate levels of the two strategies. Restif and Koella (2004) showed that resistance and tolerance can be mutually exclusive, interchangeable, or complementary components of a mixed strategy of defense, depending on the shape of the costs of resistance and tolerance. They advocated that resistance and tolerance should be regarded as complementary strategies that have different effects at individual, demographic, or epidemiological scales. However, they indicate that very little is known about the actual shapes of the cost functions in natural systems (Restif and Koella, 2004).

A second theory is based on the conceptual basis of life history theory, i.e., the notion that immune systems are costly to produce, run, and maintain, and will therefore trade off against other life history traits. For example, it is hypothesized that species that develop quickly with rapid growth and short life spans invest relatively little in defenses but favor investment in growth and early reproduction, whereas species that develop slowly, with more gradual growth and longer life spans and therefore with a higher likelihood of parasite encounter, invest more resources into costly defenses (Johnson et al., 2012). Indeed host traits such as body size, development time, clutch size, lifespan, and morphology have been found to correlate with host parasitemia or immunological defenses in birds, mammals, humans, plants, and reptiles (Johnson et al., 2012). Results by Lee et al. (2008) support the hypothesis that bird species with fast life histories have immune defenses that are characterized by an emphasis on developmentally inexpensive innate constitutive defenses despite the high costs when activated (Lee, 2006). Adults of fast living species rely more heavily on rapidly developed complement proteins (a constitutive component of the innate immune system), than adults of slow-living species who utilize antibody-mediated immune defenses (a component of adaptive immune defense) more heavily. Individuals of slow living species presumably face a greater number of infections overall and are more likely to encounter the same pathogen multiple times. Because adaptive immunity tends to have lower costs of use, high natural antibody titres may allow slow-living species to reduce the immediate costs of pathogen exposures (Lee et al., 2008).

Both tolerance and resistance traits require (re)allocation of resources and carry physiological costs (Møller et al., 1998; Roy and Kirchner, 2000), but the correlations of resistance and tolerance with other life-history traits may be different (Restif and Koella, 2004). The evolved function of an immune response is to protect an individual from harm caused by a pathogen which may be measured and defined not only immunologically, but also functionally. It is generally assumed that a strong immune response (i.e., pathogen resistance, e.g., higher antibody titres) is better than a weak immune response as animals in such a case are said to be immunosuppressed, immunocompromised, less immunocompetent or even immune-incompetent. However, achieving optimal fitness in a particular environment does not necessarily mean all fitness traits are expressed at their optimum (Allen and Little, 2011). From a cost/benefit perspective, a partially effective immune response can achieve the greatest fitness benefits. For example, where “more immunology” may result in immunopathology, the cost of eliminating or preventing the infection (resistance) may outweigh the cost of living with the infection (tolerance) (Hanssen et al., 2004; Viney et al., 2005). It is therefore conceivable that the cost to the individual of responding to infection (expenditure of metabolic resources, host-induced pathology, and compromised response to other parasite species) may favor a selective advantage of a more moderate response and tolerance (Behnke et al., 1992; Zuk and Stoehr, 2002). Or as Kogut (2009) states: “an optimal immune response to an infection might not be fully immunocompetent but would be immunosufficient or immunoresponsive.”

The two theories were combined in the work of Jokela et al. (2000), who hypothesized that effectiveness of different defense mechanisms by the host is closely linked to the diversity of attack types by the enemies resulting from ongoing coevolution between hosts and enemies. In the presence of pathogens and parasites, a high diversity of attack mechanisms by the enemy inherently reduces the effectiveness of defense by the host; as effectiveness of defense decreases, the optimal allocation of resources to defense may flip from resistance to tolerance mechanisms (Jokela et al., 2000). In addition, optimal immune function is not required for survival under most circumstances such that fitness may be lowered in defended individuals in the absence of enemies (Jokela et al., 2000; Segerstrom, 2007). This is supported by a study by Sawalha et al. (2007), who showed that in sheep, PrP genotypes associated with higher susceptibility to scrapie are associated with improved postnatal survival in the absence of the disease which indicates that this susceptibility allele has selective superiority in the absence of infection. Modeling by Doeschl-Wilson et al. (2009) indicates that unfavorable associations of the scrapie resistant PrP haplotypes with post-natal lamb mortality can increase scrapie prevalence during an epidemic, and result in scrapie persisting in the population. In the study of Kraaijeveld and Godfray (2008), after 15 generations of selection for resistance to a fungal pathogen in *Drosophila melanogaster*, selected flies had lower fitness than control flies in the absence of fungal infection. If resistance depends on possessing the machinery necessary to mount a defense should infection occur, then counter-selection in the absence of the pathogen is likely in favor of tolerance mechanisms (Jokela et al., 2000; Zuk and Stoehr, 2002).

## Resource intake

Life history patterns result from expenditure of energy and specific nutrients on fitness-related activities (Boggs, 1992; Ricklefs and Wikelski, 2002; Rauw, 2009). If the sum of energy expenditure does not match the energy intake, the balance is buffered by the storage capacity of the system. In the long-run, however, energy intake must balance energy expenditure (Weiner, 1992). Infection results in the disruption of normal processes of nutrient intake, digestion, and absorption (Lochmiller and Deerenberg, 2000). The nutritional responses during a generalized infection include alterations in rates of protein synthesis and degradation, fatty acid and carbohydrate metabolism, and alterations in the metabolic processing of individual amino acids, electrolytes, minerals, trace elements, and vitamins (Beisel, 1977). There is a particular emphasis on the ability of host tissues to manufacture specific key proteins in sufficient quantity since both the immune response (pathogen resistance, including lymphocyte proliferation, antibody production, and cytokine release) as well as the repair of cellular and tissue damage (pathogen tolerance) are all dependent upon protein-synthesizing mechanisms (Beisel, 1977; Lochmiller and Deerenberg, 2000). Certain types of proteins are synthesized at accelerated rates, whereas many individual amino acids may be wasted to accelerated processes of, for example, gluconeogenesis (Beisel, 1977; Le Floc'h et al., 2004). The acceleration of protein catabolism results in protein malnutrition and wasting of body tissue; protein malnutrition is instilled in a few days while this would take several weeks to develop during simple starvation (Beisel, 1977; Lochmiller and Deerenberg, 2000; McDade, 2005).

The sharply negative body nitrogen balance is exacerbated by a marked reduction in dietary food intake during the period of acute illness, although nitrogen may be lost from the body without the absence of a diminished dietary intake (Beisel, 1977). One of the earliest responses to infection is cytokine-mediated anorexia, where interleukins 1, 6, and 8, tumor necrosis factor and interferon alpha are released by the host defense mechanisms resulting in reduced nutrient intake through effects on the central nervous system (Donabedian, 2006). The immune system does not have to be challenged to a great degree to alter nutrient dynamics in the host because even rather mild immune reactions, like those associated with vaccination, can suppress feed intake and development (Lochmiller and Deerenberg, 2000). Because infection-induced reduction in food intake seems paradoxical during a period of high energy expenditure, traditionally, anorexia was thought of as an adverse secondary response to infection that served no function to the host. However, since this response is common among animals, it is now hypothesized that anorexia might rather be an adaptive trait that modulates the host's ability to fight infection (Ayres and Schneider, 2009). Kyriazakis et al. (1998) proposed that anorexia during parasitic infection is an evolved adaptation of the host for promoting an effective immune response and for becoming more selective in its diet toward foods that either minimize the risk of infection or are high in antiparasitic compounds. Anorexia means that demands for amino acids to support immune activation must be met from the proteins stored in the body. However, the amino acid pattern required to support immune response is different from that released by skeletal muscle proteolysis, resulting in an excess of non-limiting amino acids, whereas others become limiting for immune response. This internal amino acid balance in which the supply of muscle protein does not match the demand results in tissue loss and eventually malnutrition (Reeds and Jahoor, 2001; Le Floc'h et al., 2004).

The influence of malnutrition on resistance to infection is well established since it is the primary cause of immunodeficiency in humans worldwide (e.g., Tomkins, 1986; Katona and Katona-Apte, 2008; Panda et al., 2010). Several studies, but mostly in ruminants, have investigated the influence of nutrition and dietary manipulation on the ability of the host to cope once infected. According to Coop and Sykes (2002), evidence in the literature supports the view that protein supplementation has little or no effect on the ability of young growing livestock to prevent the early establishment of a parasite infection, however, the major effect of protein appears to be on the speed or degree to which the animal can express immunity against an established parasitic challenge. Van Houtert et al. (1995) and Butter et al. (2000) observed that worm egg concentrations in faeces were significantly reduced and apparent rate of worm expulsion considerably increased when sheep where given protein supplementation while infected with *Trichostrongylus colubriformis.* Likewise, dietary crude protein content decreased faecal worm egg counts significantly after infection with Haemonchus contortus in the study of Datta et al. (1998). The literature review by Knox and Steel (1996) concluded that low cost supplements, which supply nitrogen and essential minerals, will reduce the effects of parasitic infection in small ruminants by increasing weight gain and wool production and reducing faecal egg output and parasite burden. Sykes and Coop (2001) state that both resistance of sheep to larval establishment and performance during larval challenge can be enhanced by improved protein nutrition. In addition to protein, several other nutrients are known to influence immune functions, including vitamins, minerals, and fatty acids, therefore, in theory, scarcity of any of these nutrients may cause reduced resistance to infection to some extend (Houdijk et al., 2001).

Kyriazakis et al. (1994) observed that sheep infected daily with a small number of larvae of the small-intestinal parasite *T. colubriformis* are actually able to choose a diet high in protein content in order to meet the increased protein requirements resulting from such an infection. Similar results were found in larvae of caterpillars *(Spodoptera littoralis)* experimentally challenged with a highly virulent entomopathogen (nucleopolyhedrovirus) in the study of Lee et al. (2006) and in larvae of the African armyworm *(Spodoptera exempta)* experimentally infected with an opportunist bacterium *(Bacillus subtilis)* in the study of Povey et al. (2009). Both studies showed that infected larvae selected diets with higher levels of protein relative to uninfected larvae when offered a higher protein diet choice. In the widest sense, successful diet selection can be described as self-medication, with animals choosing a greater or lesser proportion of a food in order to match its optimum intake to defend itself against an illness (Forbes, 2007). Specific amino acid requirements need to be taken into account in order to preserve muscle mass and performance of farm animals (Le Floc'h et al., 2004).

## Resource allocation and trade offs

### Resource priority and homeorhesis

Organisms can be thought of as being informed resource users which have evolved diverse resource management systems to cope with a variety of challenging environmental conditions (Glazier, 2009a). Because of limited and variable availability of resources, organisms have evolved priority systems for allocating resources to various activities and structures in a hierarchical fashion (Glazier, 2009b). Some organ systems, such as the brain and the heart, have high energetic priority at all times, whereas others, including the immune system, can be spared when necessary (Segerstrom, 2007). In addition, there may be good adaptive reasons for not overlapping different life-cycle stages, such that control mechanisms may constrain certain combinations of physiological, behavioral and anatomical states from occurring together (Ricklefs and Wikelski, 2002). There is abundant evidence indicating that at different stages of the life cycle various metabolic pathways are up- or down-regulated resulting in nutrients that are divided in various amounts to different tissues, biological functions and end products (Collier et al., 2009). This change in tissue responses to homeostatic controls is called homeorhesis, which represents “the orchestrated or coordinated changes in metabolism of body tissues to support a physiological state” (Bauman and Currie, 1980; Collier et al., 2009). Homeorhesis was initially extensively described for the physiological state of lactation where marked alterations in the partitioning of nutrients and metabolism of the animal occur to accommodate the demands of the mammary gland. In addition, the preference of other body tissues for nutrients is altered to allow partitioning of a greater percentage of glucose to the mammary gland (Bauman and Currie, 1980). Meanwhile, the general concept of homeorhesis has been extended to include many other physiological states, nutritional and environmental conditions and pathological states as summarized in Collier et al. (2005). Also infection elicits a complete shift in metabolic priorities within the host to those associated with immunity (Lochmiller and Deerenberg, 2000; Le Floc'h et al., 2004). Spurlock (1997) discussed the physiological processes that take place during periods of immune challenge, in which pro-inflammatory cytokines orchestrate a homeorhetic response directing nutrients away from tissue growth in support of immune function. This cytokine-mediated “reprogramming” of nutrient uptake and utilization ensures an adequate supply of nutrients for proliferation of lymphocyte and macrophage populations, antibody production, and hepatic synthesis of acute phase proteins (Spurlock, 1997). A study by DiAngelo et al. (2009) in *Drosophila melanogaster* suggested that activation of the Toll signaling pathway in fat suppresses insulin signaling, leading to a decrease in nutrient stores and growth. The authors suggest that communication between these regulatory systems evolved as a means to divert energy in times of need from organismal growth to the acute requirement of combating infection.

Maintenance (survival or longevity) is usually given precedence over growth and reproduction when animals are given limited food, or are stressed in other ways as this will guarantee survival in the short term (Kyriazakis and Houdijk, 2006; Glazier, 2009a). For this reason, maintenance functions are relatively insensitive to (moderate) changes in nutrient supply. Traditionally, immune functions have been regarded as part of maintenance; however, evidence shows that at least some aspects of immunity are sensitive to changes in nutrient intake (Coop and Kyriazakis, 1999). When resources are limited, in some situations it could be adaptive for organisms to direct energy away from the immune system toward protecting and restoring other functions which may manifest itself in the form of tradeoffs (Segerstrom, 2007).

McNamara and Buchanan (2005) hypothesize that under stressful conditions animals must allocate their limited resources between the competing demands of combating the stressor (resistance) and maintaining condition (tolerance). Increasing allocation of resources to combating the stressor will leave fewer resources for adequate maintenance, increasing the chance of mortality due to the build-up of damage. This is also suggested by Segerstrom (2007) who hypothesized that energy used by the immune system represents a lost opportunity to spend that energy remediating resource loss and resolve other demands. According to the model by McNamara and Buchanan (2005), in a situation of resource restriction, the optimum strategy for resource allocation to combating an immediate physiological threat depends on the cost to individual condition and the threat and duration of the stress period. The optimal strategy concerning the immune system will depend on the pathogenicity of the environment as well as on the body condition and the costs and success of mounting an immune response (Lochmiller and Deerenberg, 2000).

Speakman (2008) suggested that the reduced immunocompetence observed during lactation may not be a compensatory cost resulting from diverting resources away from immunocompetence toward lactation, but a consequential cost resulting from a reduction in fat content and subsequent changes in circulating levels of leptin. Leptin directly influences immune cells, stimulating T-cell immunity, phagocytosis, cytokine production and haemopoiesis, resulting in attenuated susceptibility to infection. French et al. (2007) termed this the “obligate regulation hypothesis,” where immune function will be suppressed in all reproductive animals regardless of energetic state because of circulating hormone concentrations. For example, the action of sex steroids may influence both reproduction and immune function. However, since food availability does have a profound effect on immune function, they rejected this hypothesis and supported instead the “facultative regulation hypothesis” which states that energy resource availability is the driving force behind the context-dependent relationship between reproductive and immune systems, with functional trade-offs only occurring when resources are limited.

Discrepancies between studies investigating trade-offs may be a result of differing resource availability because energy conflicts may only manifest during resource-intensive times (French et al., 2007). This is supported by work of Doeschl-Wilson et al. (2009), who showed in a mathematical model that the relationship between a host's response to pathogen challenge and production potential largely depends on the interaction between its genetic capacity for production and disease resistance with the nutritional environment. The observation that selection for high production efficiency has resulted in several undesirable side effects that are mostly related to metabolic imbalance, i.e., a mismatch between increased output (selection for high production) and decreased input (selection for increased feed efficiency and reduced body fat reserves), suggests that we can expect our farm animals to be restricted by their environment (Rauw, 2009). Trade-offs may not be found at all if two processes do not share important resources, if resources are not limited or if the trade-off does not involve the immune parameter being measured (Lee, 2006). In addition, several parts of the defense mechanisms may not incur significant fitness costs (Coustau et al., 2000; Rigby et al., 2002).

### Trade-off between immune function and growth

The negative influence of activation of the immune system on growth is well established resulting both from a reduced feed intake through anorexia and from redirection of resources toward an immune response away from other functions. For example, chronic immune stimulation in non-vaccinated sows that were farrowed in a non-sanitized farrowing room and that did not receive antibiotics resulted in reduced body weight gains in pigs in the study of Williams et al. (1997). Immune challenge with *E. coli* lipopolysaccharide resulted in reduced weight gain in weanling pigs in the study of Van Heugten and Spears (1997). Mauck et al. (2005) observed an inverse relationship between growth rate and the development of components of the avian immune system in a wild population of Leach's Storm-Petrels (*Oceanodroma leucorhoa*), although this trade-off was suggested to be more complex than resulting from simple energy allocation. Daily injections of the inflammatory agent Sephadex resulted in significantly lower rates of weight gain in chicks in the study of Klasing et al. (1987). Reciprocally, in the study of Allen and Little (2011), stimulating an increased development rate in juvenile *Daphnia* resulted in an increased infection rate when exposed to the parasite *Pasteuria ramosa*, suggesting that allocation of resources to development left the fish lacking in ability to allocate an adequate amount to parasite resistance. Coop and Kyriazakis (1999) theorized that growing animals that encounter parasites for the first time can be expected to prioritize resources to the acquisition of immunity over growth, whereas once immunity has been acquired, growth and reproduction would be prioritized over expression of immunity to parasites. Indeed, a large body of evidence shows that increased metabolizable protein supply reduces fecal egg counts and worm burdens in ruminants only at later stages in experimental parasitic infestations, which supports this view that acquisition, but not expression, of immunity takes priority over growth (Houdijk and Athanasiadou, 2003).

### Trade-off between immune function and lactation

Reproductive effort, and in particular lactation, is a resource-prioritized process that requires substantial energy and other nutrient resources. The prevalence and intensity of parasitic infection often increases in animals when they are reproducing, which may result from adaptive reallocation of resources in times of increased energetic demand (Deerenberg et al., 1997). Increased brood size resulted in a reduced probability of detecting any immune response against sheep red blood cells in zebra finches (*Poephila guttata*) in the study of Deerenberg et al. (1997). Verhulst et al. (2005) suggest that zebra finches *(Taeniopygia guttata)* rearing large broods have lower antibody responses because they economize on the maintenance costs of the immune system. Furthermore, in collared flycatchers (*Ficedula albicollis*), increased brood size resulted in reduced antibody production when immunized with Newcastle disease virus in the study of Nordling et al. (1998), and in reduced T-cell-mediated immune response when injected with phytohemagglutinin in the study of Moreno et al. (1999). Breeding grey partridges (Perdix perdix) immune challenged with Newcastle disease virus laid smaller eggs, suggesting that immune challenge can have physiological consequences in terms of self-maintenance and reproductive allocation to the egg (Cucco et al., 2010).

In several species of mammals, an increasing number of experimental studies indicate that competition for nutrients between the immune system and reproductive effort may result in a peri-parturient breakdown of acquired immunity to parasites (Houdijk et al., 2001). Lactating ewes show an increased fecundity of parasites present and an inhibition of the expulsion of established parasites, while prevention or premature termination of lactation results in the expulsion of established parasites and rejection of newly acquired infection (Shubber et al., 1981). Lactating bighorn ewes had greater faecal counts of lungworm larvae compared with non-lactating females, suggesting that reproduction resulted in a decrease in resistance to parasites and pathogens (Festa-Bianchet, 1989). Ewes that have acquired immunity to nematode infection tend to lose it around the time of parturition and during lactation, and strains of sheep selected for resistance to nematode infection still undergo a peri-parturient loss of immunity (Barger, 1993). However, Xu et al. (2012) showed that immune function is not suppressed to compensate the high energy demands during lactation in Brandt's voles.

### Trade-off between immune function and stress response

Trade-offs may result from resources that are allocated to deal with external stresses (Svensson et al., 1998). The stress response includes metabolic, energetic, immune, endocrine, neural, and behavioral changes that are aimed at overcoming the stressful situation and compensating for the imbalances produced by the stressor (Selye, 1953; Tort, 2011). Stress, through the action of stress hormones such as glucocorticoids, catecholamines, prolactin, growth hormone and nerve growth factor, has detrimental effects on immune function (Moberg, 2000; Webster Marketon and Glaser, 2008). Cortisol simultaneously makes more glucose available from energy stores and suppresses certain physiological activities such as immune activity and reproduction (Segerstrom, 2007). In addition, the consequences of stress include elevated metabolic costs since energy is needed by the animal to cope with the stress.

The stress model developed by Moberg (2000) explains the concept of trade-offs between stress and other functions. An animal has a budget of resources that are available to service basal biological functions, in addition, the animal has available a reserve from which it must draw to deal with stress. The biological cost of stress depends on the duration of the stress (acute vs. chronic), the severity of the stressor, and on the number of stressors (or repeated exposure to the same stressor). When the biological cost is met by the reserves, the stressor will have no impact on the other biological functions; however, when there are insufficient biological reserves available, resources must be reallocated away from other biological functions that now become impaired. At this time the animal enters a pre-pathological-pathological state due to a reduction in its physiological state, and experiences distress (Moberg, 2000).

Environmental stressors are involved in the aetiology of important livestock diseases, including transmissible gastroenteritis in young pigs, Newcastle and Marek's disease in chickens and shipping fever in cattle (Kelley, 1980). In an extensive review, Kelley (1980) identified eight stressors that typically occur in modern livestock production systems: heat, cold, crowding, mixing, weaning, limit-feeding, noise, and restraint and all of these stressors have been shown to alter the immune system of animals. Effects of stress on immune function in fish have been reviewed by Tort (2011). When the stressor is acute and short-term, the response pattern is stimulatory and the fish immune response shows an activating phase that specifically enhances innate responses; however, if the stressor is chronic, the immune response shows suppressive effects and therefore the chances of an infection may be enhanced (Tort, 2011). In humans, acute stressors enhance low-energy-consuming immune components and suppress high-energy-consuming ones, whereas stressors lasting from days to years are associated with suppression of a number of different immune functions, including protein production, cell production, and cell function (Segerstrom, 2007). Strenuous stress also tends to suppress several aspects of immune function and, vice versa, costly behaviors are reduced in animals mounting an immune response (Svensson et al., 1998; Viney et al., 2005). Sickness behavior that is characterized by increased fatigue, sleep, withdrawal and a decreased interest in pleasurable behaviors is initiated by the host as a result of activation of the immune system (Segerstrom, 2007).

### Consequences of selection for increased production on immune function and vice versa

Genetic selection has increased production levels of livestock species considerably; however, animals in a population that have been selected for high production efficiency appear to be more at risk for behavioral, physiological, and immunological problems (Rauw et al., 1998). Artificial selection may result in preferential allocation of resources to the traits selected for, leaving animals lacking in ability to respond adequately to other demands. In particular those traits that are not specifically included in the breeding goal may be affected, i.e., traits other than production traits, because their importance is not specifically recognized (Rauw, 2009).

Genetic selection of poultry for superior growth rate may result in decreased resistance to disease or reduced immunological response (Bayyari et al., 1997). A meta-analysis by Van der Most et al. (2011) indicated that selection for accelerated growth in poultry had a large and significantly negative effect on immune function. Chickens from a line selected for faster growth were more susceptible to the development of Marek's disease than chickens from a line exhibiting a slower growth rate in the study of Han and Smyth (1972). Broilers selected for high growth rate showed lower antibody responses when challenged with sheep erythrocytes (SRBC) than animals from a low body weight line (Miller et al., 1992) and a randombred control line (Qureshi and Havenstein, 1994). Koenen et al. (2002) conclude that fast growing broiler chickens are specialized in the production of a strong short-term humoral response, whereas slow growing layer-type chickens are specialized in a long-term humoral response in combination with a strong cellular response, which is in conformity with their life expectancy. In the study of Saif et al. (1984), a natural outbreak of erysipelas and fowl cholera resulted in a higher mortality rate in turkeys from a line selected for increased growth rate than in animals from an unselected control line. Mortality of turkeys from the selected line was higher than that of animals from the unselected control line when subsequently experimentally challenged with *Pasteurella multocida* (Sacco et al., 1991; Nestor et al., 1996a,b) or with Newcastle disease virus (Tsai et al., 1992). In addition, animals from the fast growth line had a lower toe web response to phytohemagglutinin-P, lower lymphocyte counts, and lower relative spleen weights than animals from the randombred control line (Bayyari et al., 1997). In mice, Coltherd et al. (2009) concluded that artificial selection for high growth may reduce the ability to cope with pathogens and that improved protein nutrition may to some extent ameliorate this penalty.

In dairy cattle, overall, there is clear evidence that there are negative genetic associations between milk yield and health (Veerkamp et al., 2009). Clinical mastitis cases are principally associated with one of the following bacteria: *S. Aureus, E. Coli, Streptococcus dysgalactiae, Streptococcus uberis, CNS, Arcanobacterium pyogenes*, or *Klebsiella spp* (Rupp and Foucras, 2010). The genetic antagonism between milk yield and mastitis resistance has been well established (Rupp and Boichard, 2003). The average genetic correlation between milk yield and mastitis was reviewed to be 0.30 across seven studies by Emanuelsson (1988), 0.38 across 16 studies by Pryce et al. (1997), and 0.43 in Nordic data by Heringstad et al. (2000). After four generations of selection for milk production in a divergent selection experiment in dairy cattle, the genetic difference in mastitis between the high and low milk production group was 3.1% clinical mastitis as a correlated response (Heringstad et al., 2003). Although studies are rare for goats and sheep, they do confirm the positive relationship between milk yield and measurements of mastitis (Raynal-Ljutovac et al., 2007). Rupp and Boichard (2003) suggest that pleiotropic genes could be involved, but also biological competition for energy and nutrients between functions.

Romney sheep selected for increased fleece weight had higher feacal worm egg counts (Howse et al., 1992); Eady et al. (1996) estimated that genetic selection for productivity in sheep would lead to a 1% per annum increase in feacal worm egg counts. In Australia and New Zealand, egg counts following nematode infection are unfavorably correlated with wool growth and live-weight; however, these correlations are consistently favorable in Europe (Stear et al., 2001).

Reciprocally, divergent selection for sheep red blood cell antibody response in a White Leghorn population resulted in reduced body weight in the studies of Gross et al. (2002) and Lamont et al. (2003). Martin et al. (1990) observed that females from the low line were heavier as juveniles but lighter as adult, matured at a younger age, and had higher egg production than those from the high line. In the study of Lamont et al. (2003), a difference in body weight was observed as early as 7 days after hatch; after 20 generations of selection, animals from the line selected for high antibody response were 20% lighter and matured 30 days later than animals from the line selected for low antibody response. Selection for resistance to Marek's disease in chickens resulted in animals with lower adult body weight and smaller eggs than animals from unselected lines (Warner et al., 1987).

Selection for reduced helminth feacal egg counts may result in lower lamb growth rates (Bisset et al., 2001). In the study of Morris et al. (2000), selection for low feacal worm egg count in Romney sheep resulted in decreased post-weaning weight gain and decreased fleece weight in yearlings and ewes. Tendencies toward unfavorable relationships between immune-competence and lean growth capacity have been reported in growing pigs (Knap and Bishop, 1996). The genetic trend for protein yield after four generations of selection for milk production in a divergent selection experiment in dairy cattle was significantly negative in a line selected for low clinical mastitis, corresponding to −1.97 kg protein per cow per generation (Heringstad et al., 2003).

## Trade-offs with immune tolerance

The trade-offs described above between production traits and immune function may be mostly ascribed to immune resistance, although immune tolerance mechanisms such as damage repair may have been involved. Trade-offs with immune tolerance seems to be difficult to consider because of the difficulty in separating the processes involved in damage repair from other cell maintenance functions, in addition, literature on immune tolerance in animals is scarce. Immune tolerance is correctly evaluated by measuring the fitness response to a gradient in intensity of infection (Simms, 2000), and such data is not yet available. Trade-offs between protein turnover and production traits have been described in non-immune challenged animals. For example, selection for increased growth rate has resulted in slower protein turnover rates and reduced energy requirements for maintenance in rats (Bates and Millward, 1981), chickens (Thomas et al., 1991), lambs (Oddy et al., 1995) and cattle (Richardson and Herd, 2004). Increasing the degree and/or effectiveness of cell and tissue maintenance functions with selection for immune tolerance can be expected to result in higher energy and protein expenditures and consequently trade-offs with other economically important production traits.

However, improving tolerance mechanisms may have positive consequences for overall adaptability and robustness. For example, protein turnover provides the flux that is necessary for metabolic regulation and adaptation. It enables the metabolic adjustments required for maintenance of homeothermy, reproduction, and development, the repair of damaged tissue, maintenance of the immune system in a state of readiness, combating infection, and during or following changes in the environment or in the nutritional/physiological status (Hawkins, 1991; Lobley, 2003). Pirlet and Arthur-Goettig (1999) suggest that the evolution of life results from specific degradation of defective, old, damaged, denatured protein molecules, which forces the selection of structurally superior proteins. Protein turnover is furthermore involved in the ageing process, in the maintenance and error correction of functional proteins through the removal of proteins damaged by oxidative stress (Tavernarakis and Driscoll, 2002). Hawkins (1991), in an excellent review, indicates that intense whole-body protein turnover may enhance viability by enabling the metabolic adjustments necessary for regulation and adaptation. Faster protein turnover may enhance performance by improvement of sensitivity in metabolic and endocrine control, facilitating faster acclimation in the regulation of metabolic flux, as well as functioning in the mobilization and selective redistribution or catabolism of amino acids, elimination of non-functional or denatured polypeptides, and thermogenesis. Thus, improved protein turnover rate may improve the ability of an animal to adapt to new dietary and physiological conditions in addition to immune tolerance, i.e., improve robustness (Baldwin et al., 1980).

Phenotypic changes across environments for a wide variety of different characters in plants and animals, in natural and agricultural systems, and over both temporal and spatial variation in the environment is the basis of “phenotypic plasticity” which is determined by the shape of the reaction norm of the phenotypic values expressed by a genotype across a range of environments (Via et al., 1995). Plant ecologists have adapted the method to deal with questions of resistance vs. tolerance to pathogens with reaction norms that relate host health to infection intensity. Resistance is a measure of the ability of a host to limit pathogen growth and thereby maintain health, which can be interpolated as the inverse of the mean of the pathogen load. Tolerance is a measure of the ability of a host to survive an infection at a given pathogen load, which is represented by the slope of the curve (Simms, 2000; Schneider and Ayres, 2008). Thus, improving resistance would consist of moving the animal up the reaction-norm curve toward a lower pathogen load and higher health, whereas improving tolerance would entail flattening the slope of the curve. Råberg et al. (2007) conclude that this method is readily transferable to domestic animals where it could be used to work out optimal selection strategies to enhance immune defense mechanisms.

A tolerant genotype minimizes the decline in fitness from that achieved in a relatively benign environment to that in a relatively stressful environment; thus, measuring tolerance involves measuring fitness in more than one environment (Simms, 2000). In essence, selection for immune tolerance in farm animal species is a particular case of selection for animal robustness. Robustness is defined by Knap (2005) as “the ability to combine a high production potential with resilience to stressors, allowing for unproblematic expression of a high production potential in a wide variety of environmental conditions.” Two options for breeding for animal robustness are extensively described by Knap (2009): the direct approach involves the inclusion of directly measurable robustness traits in the breeding objective and in the selection index, whereas an indirect approach involves the use of reaction norms analysis to estimate breeding values for the environmental sensitivity of the genetic potential for production performance. Reaction norms are a measurement of the phenotypes for a given genotype across a range of environments that measure how an individual responds to a range of environmental conditions (Schneider and Ayres, 2008). In animal production this means that progeny of sires are spread across a wide environmental range and are recorded for the production traits of interest. The production performance is then regressed on a descriptor of the environment (from a worse to a better environment, production is expected to increase) where animals with high resilience to external stressors (i.e., animals with a flatter slope) will be more robust and hence more desirable (Friggens and Van der Waaij, 2009; Knap, 2009). Because of the increasingly wide variety of environmental conditions in which livestock animals are required to perform, and evidence that expression of high production potential is more compromised in high producing animals, robustness has a high priority in current livestock production. As Mormède et al. (2010) state: the farm animal of the future is robust, adapted and healthy. Therefore, a possible relationship between immune tolerance mechanisms and other robustness traits would be highly desirable.

## Conclusions: selection for resistance or tolerance?

Breeding for immune defenses is needed to improve sustainability of livestock systems and is becoming more common throughout the world (Stear et al., 2001; Bishop et al., 2002). The distinction between resistance and tolerance is of importance since it determines the suitability of selection for different disease scenarios (Bishop et al., 2002). Both are genetically determined by many genes that indicate that selective breeding is feasible; however, both resistance and tolerance are life history traits that require (re)allocation of resources and carry physiological costs which may trade off against other economically important traits when resources are limited.

Stear et al. (2001) raise several concerns about the desirability of breeding for disease resistance. One concern is that there may be unfavorable consequences for other diseases; for example, when selective breeding for resistance to a specific disease may predispose hosts to prefer one class of immune response, leaving them susceptible to infectious agents that are normally controlled by another type of response. A counter argument is that selective breeding for resistance to immunosuppressive diseases would reduce the prevalence of these diseases and enhance overall immune responsiveness (Stear et al., 2001). In addition, it may be possible to select for resistance to several diseases by selecting for enhanced immune responsiveness (Wilkie and Mallard, 1999; Stear et al., 2001).

As outlined in the previous section, mechanisms involved in disease tolerance (damage repair) appear to be of a more general nature. In addition, mechanisms of cell maintenance and repair may be involved in adaptability to new nutritional, physiological and environmental conditions, i.e., animal robustness. Selection for increased production efficiency has narrowed the amount of resources that are available to the demands of maintenance, growth and reproduction. This reduction in metabolic space may reduce an animal's resilience to stressors and its ability to adapt to a wide variety of environmental conditions. Therefore, breeding goals that include robustness traits are required in the implementation of more sustainable agricultural production systems (Knap, 2009; Rauw, 2012). They combine robustness traits with production traits, balancing production potential with environmental sensitivity; this will increase or restore the animals' ability to interact successfully with the environment and improve welfare and productivity (Knap, 2009). It will be therefore of great interest to investigate the theory that immune tolerance is a robustness trait that may be positively correlated with overall animal robustness.

Considerably more research is needed to estimate the shapes of the cost functions of different immune strategies, and investigate trade-offs and cross-over benefits of selection for disease resistance and/or disease tolerance in livestock production.

### Conflict of interest statement

The author declares that the research was conducted in the absence of any commercial or financial relationships that could be construed as a potential conflict of interest.
